# Predictors of Utilisation of Skilled Maternal Healthcare in Lilongwe District, Malawi

**DOI:** 10.15171/ijhpm.2019.67

**Published:** 2019-08-13

**Authors:** Isabel Kazanga, Alister C. Munthali, Joanne McVeigh, Hasheem Mannan, Malcolm MacLachlan

**Affiliations:** ^1^School of Public Health and Family Medicine, College of Medicine, University of Malawi, Blantyre, Malawi.; ^2^Centre for Social Research, Chancellor College, University of Malawi, Zomba, Malawi.; ^3^Department of Psychology, Maynooth University, Maynooth, Ireland.; ^4^Assisting Living and Learning (ALL) Institute, Maynooth University, Maynooth, Ireland.; ^5^School of Nursing, Midwifery and Health Systems, University College Dublin, Dublin, Ireland.; ^6^Centre for Rehabilitation Studies, Stellenbosch University, Cape Town, South Africa.; ^7^Olomouc University Social Health Institute, Palacký University, Olomouc, Czech Republic.

**Keywords:** Malawi, Health System, Maternal Healthcare

## Abstract

**Background:** Despite numerous efforts to improve maternal and child health in Malawi, maternal and newborn mortality rates remain very high, with the country having one of the highest maternal mortality ratios globally. The aim of this study was to identify which individual factors best predict utilisation of skilled maternal healthcare in a sample of women residing in Lilongwe district of Malawi. Identifying which of these factors play a significant role in determining utilisation of skilled maternal healthcare is required to inform policies and programming in the interest of achieving increased utilisation of skilled maternal healthcare in Malawi.

**Methods:** This study used secondary data from the Woman’s Questionnaire of the 2010 Malawi Demographic and Health Survey (MDHS). Data was analysed from 1126 women aged between 15 and 49 living in Lilongwe. Multivariate logistic regression was conducted to determine significant predictors of maternal healthcare utilisation.

**Results:** Women’s residence (*P*=.006), education (*P*=.004), and wealth (*P*=.018) were significant predictors of utilisation of maternal healthcare provided by a skilled attendant. Urban women were less likely (odds ratio [OR] = 0.47, *P*=.006, 95% CI = 0.28–0.81) to utilise a continuum of maternal healthcare from a skilled health attendant compared to rural women. Similarly, women with less education (OR = 0.32, *P*=.001, 95% CI = 0.16–0.64), and poor women (OR = 0.50, *P*=.04, 95% CI = 0.26–0.97) were less likely to use a continuum of maternal healthcare from a skilled health attendant.

**Conclusion:** Policies and programmes should aim to increase utilisation of skilled maternal healthcare for women with less education and low-income status. Specifically, emphasis should be placed on promoting education and economic empowerment initiatives, and creating awareness about use of maternal healthcare services among girls, women and their respective communities.

## Background

Throughout pregnancy and childbirth and after delivery, healthcare services are essential for the well-being, and potentially the survival, of both mothers and children, and result in significant reduction in maternal and neonatal mortality.^1,2^ Antenatal care (ANC) and a skilled health attendant during delivery are therefore crucial for reducing preventable maternal deaths.^3^ For example, in 2016, it is estimated that 78% of all live births benefitted from skilled healthcare throughout delivery.^4^ However, many women and children are dying worldwide from preventable causes and complications related to pregnancy and childbirth. Approximately 830 women die each day from preventable issues in relation to pregnancy and childbirth.^5^ Almost 100% of maternal deaths globally occur in low- and middle-income countries (LMICs), with over half of such deaths occurring in sub-Saharan Africa and almost one-third occurring in South Asia.^6,7^ Maternal mortality is a health indicator that suggests substantially wide gaps between rich and poor, urban and rural districts – between and within countries.^8^

In sub-Saharan Africa, the risk of maternal death is significantly higher due to a combination of factors including high fertility rates and lack of access to quality ANC and skilled delivery care.^3^ In Malawi, poor quality of delivery facilities, for example, is associated with higher risk of newborn mortality.^9^ However, individual factors such as education and place of residence are also critical regarding infant health in Malawi.^10^ According to the 2014 Malawi MDG Endline Survey,^11^ the highest percentages of women who do not receive ANC and postnatal care include women residing in rural areas, those with no education, and those in lower wealth quintiles; similarly, with regards to delivery care, women in urban areas, those with higher education levels, and those in the highest wealth quintile are more likely to deliver in a health facility. Indeed, several individual factors have been identified as being associated with utilisation of maternal health services, including religion,^12^ education and employment,^13-15^ ethnicity,^13,16,17^ household wealth,^13,16,18-21^ mother’s age,^16,20,22^ marital status,^23,24^ and place of residence (urban versus rural).^13,16,25^

All United Nations Member States, including Malawi, have agreed to aim for universal health coverage by the year 2030, in accordance with the Sustainable Development Goals (SDGs).^26^ The SDGs comprise 17 goals and 169 targets, which aim to achieve human rights for all, gender equality, and the empowerment of women and girls; the SDGs will guide action over a fifteen-year duration towards sustainable development in the economic, social and environmental spheres.^27^ The SDGs prioritise the need for strengthened social inclusion and efforts to reach marginalised populations.^28^ Griggs et al^29^ emphasise that all SDGs interact with each other, as they are designed to be integrated goals that are in essence interdependent. For example, an association is evident between high-quality education and better health; for instance, education can influence health instantaneously through changed behaviour or the adoption of new technologies, or on a longer-term basis through increased income, opportunities, independence and empowerment.^30^ Evidence suggests that when girls with at least a basic education become adults, they are more likely than those who are not educated to manage their family’s size in accordance with their capacities, and more likely to provide better care for their children and send them to school.^31^ Strengthening education for girls (goal 4 of SDGs) in southern Africa would therefore improve maternal health outcomes (part of goal 3); in addition to supporting poverty eradication (goal 1), gender equality (goal 5), and economic growth (goal 8).^32^ Indeed, it is estimated that, because women with an education are less likely to die during childbirth, if all mothers completed primary education, maternal deaths would decrease by two-thirds, saving 98 000 lives; while in sub-Saharan Africa, if all women completed primary education, maternal deaths would decrease by 70%, saving almost 50 000 lives.^33^

Malawi is a leader in sub-Saharan Africa regarding implementation of evidence-based policies to support maternal and child health.^9^ However, while Malawi has made significant improvements regarding child mortality, maternal and newborn mortality rates remain too high.^34^ While one of the targets for SDG 3 is to decrease the global maternal mortality ratio to below 70 per 100 000 live births,^5^ Malawi has one of the highest maternal mortality ratios globally,^35^ estimated at 439 maternal deaths per 100 000 live births.^1^ As proposed by Colbourn et al,^36^ there is a lack of accurate data regarding trends in maternal mortality. In response to this need, the aim of this study was to identify which individual factors best predict utilisation of skilled maternal healthcare in a sample of women residing in Lilongwe district of Malawi. Identifying which of these factors play a significant role in determining utilisation of skilled maternal healthcare is required to inform policies and programming in the interest of achieving increased utilisation of skilled maternal healthcare in Malawi.

Importantly, as suggested by the World Health Organization (WHO),^37^ “to improve maternal health, barriers that limit access to quality maternal health services must be identified and addressed at all levels of the health system.” However, within levels of the health system, the influence of individual factors on utilisation of maternal healthcare services may be overlooked. As suggested by Agus and Horiuchi^25^ (p. 2), “maternal and child welfare is not only related to health services provided by government and private organizations; it is also related to women as mothers including their education, economic status, culture, environment, and professional development.” While improvements to systems and structural factors may enhance utilisation of maternal healthcare services, individual factors may continue to have a significant effect on healthcare utilisation – an assertion underpinning the premise of this study.

## Methods

### Conceptual Framework

This study used Andersen’s Behavioural Model of Health Services Use as its conceptual basis. Andersen’s model is one of the most widely acknowledged multilevel conceptual frameworks that incorporate both individual and contextual determinants of utilisation of health services.^38^ Andersen’s model has been used extensively in health services research to investigate factors that lead to utilisation of health services,^38^ and to evaluate the extent to which health services are equitably distributed or accessed.^39^ The model has been used, for example, to analyse determinants and patterns of healthcare utilisation,^40,41^ and to understand health-seeking behaviors.^42^ It has also been used to assess disparities in healthcare utilisation.^43^

Aday and Andersen argue that utilisation of health services is determined by characteristics of the population at risk. These characteristics can be conceptualised as comprising 3 components, ie, predisposing, enabling, and need components. The predisposing component includes variables that describe the “propensity” of individuals to use services such as age, gender, race, and religion.^44^ The enabling component describes the “means” available to individuals for the use of services. Examples of enabling factors include income, family support, access to health insurance, and attributes of the community in which the individual lives (urban-rural settings). The need component refers to illness level, which is the most immediate cause of health service utilisation. The need for healthcare may be either that perceived by the individual or that evaluated by the delivery system.

### Study Materials

Data analysis was conducted using secondary data from the 2010 Malawi Demographic and Health Survey (MDHS).^45^ The 2010 MDHS is a nationally representative sample comprising 27 000 households and involving 24 000 female and 7000 male respondents, to provide data to policy-makers, planners, researchers, and programme managers.^45^ This study used data from the Woman’s Questionnaire of the MDHS.

The Demographic and Health Survey (DHS) comprises a wealth index. The DHS wealth index is calculated using households’ cumulative living standards, classifying households according to 5 wealth quintiles; calculated using data on households’ ownership of selected assets, including televisions and bicycles, and types of access to water and sanitation facilities.^46^ The 5 wealth quintiles comprise “poorest,” “poorer,” “middle,” “richer,” and “richest.”^47^

### Participants and Procedures

The study conducted a secondary data analysis of 2010 MDHS data from 1126 women aged 15-49 in Lilongwe district. As Malawi’s capital city, Lilongwe was chosen as a case study as it is the country’s largest and fastest urbanising city with a diversity of cultures, ethnicities, and healthcare practices, therefore reflecting the diversity at national level. The population of Lilongwe in 2017 was estimated as 1.1 million.^48^

The sample was selected using a stratified two-stage cluster design, with enumeration areas (EAs) being the sampling units for the first stage and households as a second stage of sampling, as detailed in the MDHS.^45^ The sampling frame was based on EAs of the 2008 Malawi Population and Housing Census. The sampling frame was stratified into 27 districts in the country; and within each of the districts, EAs were further stratified by urban and rural areas. A fixed number of 20 households were selected in urban and 35 households in rural primary sampling units. In the selected households, a total of 23 748 women aged 15-49 were eligible, and 23 020 women (97%) were interviewed. Among these, 1126 women were from Lilongwe, thus the sample size for this study. Figure  presents a flow diagram of the sampling process. Demographic characteristics of respondents are presented in Table 1.

**Figure:**
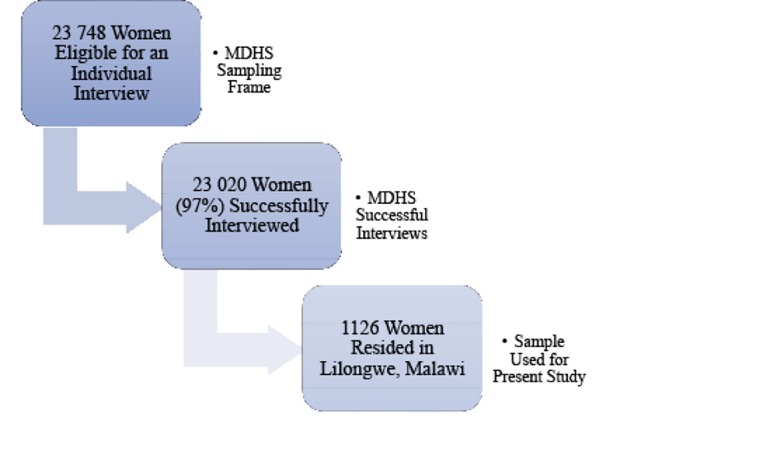
Flow Diagram of Sampling Process. Abbreviation: MDHS, Malawi Demographic and Health Survey.

**Table 1:** Demographic Characteristics of Respondents

**Variable**	**No. (%)**
Age	
15-19	243 (22)
20-34	602 (53)
35-49	281 (25)
Residence	
Urban	480 (43)
Rural	646 (57)
Ethnicity	
Chewa	831 (74)
Ngoni	112 (10)
Yao	55 (5)
Lomwe	41 (4)
Tumbuka	37 (3)
Other	33 (3)
Tonga	17 (1)
Religion	
Other Christian	405 (36)
Church of Central Africa Presbyterian	304 (27)
Catholic	236 (21)
Seventh-Day Adventist Church	79 (7)
Muslim	56 (5)
Anglican	23 (2)
No religion	23 (2)
Marital status	
Married	548 (49)
Never married	267 (24)
Cohabiting	197 (17)
Divorced/separated	80 (7)
Widowed	34 (3)
Employment status	
Employed	649 (58)
Not employed	477 (42)
Wealth	
Richest	427 (38)
Richer	169 (15)
Middle	158 (14)
Poorer	158 (14)
Poorest	214 (19)
Education	
Higher	56 (5)
Secondary	248 (22)
Primary	664 (59)
None	158 (14)

The study focused on several key questions in the MDHS in relation to use of ANC, delivery and postnatal care services. In the MDHS, women who had given birth in the 5 years before the survey were asked questions regarding their care. Specifically, women reported if they received ANC, delivery care and postnatal care from a skilled attendant (ie, doctor, clinical officer, nurse or midwife) for their most recent live birth within the 5 years preceding the survey. Furthermore, women reported the number of visits made to the ANC clinic, and the timing (trimester) of the first ANC visit during the most recent pregnancy within the 5 years preceding the survey. Respondents also reported the place of delivery during their most recent birth in the 5 years preceding the survey. Moreover, women reported the timing after delivery of their first postnatal check-up.

Permission to use the data was obtained from the DHS Programme website. Ethical approval for this study was granted by the Health Policy and Management/Centre for Global Health Research Ethics Committee at Trinity College Dublin, Ireland, and the College of Medicine Research Ethics Committee (COMREC) at the University of Malawi.

### Data Analyses

The study comprised eight independent variables, ie, age, marital status, residence (urban/rural), education, work status, economic status (wealth), ethnicity and religion. Selection of independent variables was guided by Andersen’s Behavioural Model of Health Services Use and a review of the literature. The variables were categorised according to the MDHS dataset. Dependent variables comprised utilisation of ANC, delivery care, and postnatal care by a skilled attendant.

A maternal healthcare index was created to indicate the continuum of maternal healthcare, and was generated by combining data for utilisation of ANC, delivery and postnatal care provided by a skilled attendant. The outcome variables were coded as 1 if the woman received maternal healthcare from a skilled attendant and as 0 if she did not receive maternal healthcare from a skilled attendant. The response category was collapsed to create dichotomous dependent variables with only 2 categories, ie, “yes” and “no,” on the basis of whether or not the woman had received maternal healthcare from a skilled health attendant. Variable categories with small sample sizes were combined with another category to enable meaningful analysis. For example, the sample sizes for some ethnic and religious groups were very small, and as such they were combined with others. If the respondent reported more than one person providing assistance during delivery, the most qualified person was used for analyses. Adebowale and Udjo similarly used a maternal healthcare index in their study of the relationship between infant mortality and a maternal healthcare services access index in Nigeria, using data from the 2013 Nigerian DHS; whereby their maternal healthcare services access index was created using variables including antenatal visit, antenatal attendance, tetanus injection during pregnancy, place of delivery, and birth attendance.^49^

### Descriptive Analyses

Descriptive analyses were conducted of demographic characteristics of respondents and the use of maternal healthcare services (as presented below in the Results section).

### Chi-Square Analyses

Pearson chi-square tests were conducted to explore the relationship between demographic characteristics and use of skilled maternal healthcare services. Statistical significance was established at *P* values of <.05.

### Logistic Regression

Direct logistic regression was conducted to determine predictors and factors that were significantly associated with utilisation of maternal healthcare services provided by a skilled health attendant. Analysis was conducted using IBM SPSS Statistics 22.

Multivariate logistic regression was conducted to determine predictors of maternal healthcare utilisation. Logistic models were developed using a three-step approach. The first step comprised running a series of univariate binary logistic regression models in which the relationship of each independent variable with respective dependent variables was examined. Independent variables that were significant (*P* < .1) were considered important for inclusion in the next step of the process.

The second step comprised collinearity diagnostics for independent variables chosen from the first step, as logistic regression is sensitive to multicollinearity. A cut-off value of 10 for the Variance Inflation Factor was used to determine the presence of multicollinearity.^50^ No multicollinearity was identified.

Third, binary logistic regression models were run using variables from the first step, having been assessed for multicollinearity. Models used the *ENTER* method. Of the eight independent variables, six variables (age, marital status, residence, education, work status, and wealth) were treated as potential confounders and were included in binary logistic regression models for use of skilled attendance for maternal healthcare, whether or not they had attained statistical significance in the first step. Ethnicity and religion were excluded in the regression models as potential confounders, based on a review of the literature. This stage represented the multivariate model. Statistical significance for the final models was set at *P* <.05.

The regression model was tested for ‘goodness of fit’ using the Hosmer-Lemeshow test. A good model fit is indicated by a Hosmer-Lemeshow test statistic value of *P* >.05. The Nagelkerke R Square was used as an indication of the variation in the dependent variable that could be explained by the model.^50^ Statistical significance was established at *P* values of <.05.

## Results

### Descriptive Analyses

#### Antenatal Care

In total, 91% of women obtained ANC from skilled providers, ie, a nurse or midwife (82%), and doctor or clinical officer (9%). A small proportion of women received ANC from a patient attendant (5%) and health surveillance assistant^[1]^ (1%). Less than 1% of women received ANC from a traditional birth attendant, while 3% of women did not receive any ANC services.

Among women who visited the ANC clinic, 50% visited at least four times or more during their last pregnancy. Approximately 43% of women reported 2 or 3 ANC visits, while 3% of women visited the ANC clinic only once. A total of 3% of women did not visit any ANC clinic. Furthermore, 47% of women had their first ANC visit during the second trimester (4-5 months) of pregnancy. Approximately 11% of women had their first ANC visit within the first trimester (<4 months), while 40% had their first ANC visit within the third trimester (6-7 months) of pregnancy. A total of 2% of women had their first ANC visit at 8 months or more.

#### Delivery Care

In total, 76% of deliveries occurred in a health facility, with most of the deliveries taking place in public health facilities (56%), Christian Health Association of Malawi facilities (18%), and private health facilities (2%). Approximately 23% of deliveries took place at home, while 1% occurred in other health facilities.

Most of the deliveries (76%) were conducted by a skilled attendant, ie, nurse or midwife (62%) and doctor or clinical officer (14%). A total of 3% of deliveries were assisted by a patient attendant, 16% of births were assisted by a traditional birth attendant, whereas 3% of births were assisted by a relative or friend. Approximately 1% of births were assisted by other providers, while 2% of births were not assisted.

#### Postnatal Care

A total of 34% of women did not receive any postnatal check-up. Approximately 32% of women received the first postnatal check-up within the first hour of giving birth, and cumulatively 52% of women received the first postnatal check-up within 24 hours of delivery. Cumulatively 58% of women received the first postnatal check-up 2 days after delivery. A total of 6% of women received the first postnatal check-up after 41 days subsequent to delivery.

Half of the women (50%) received the first postnatal check-up from a nurse or midwife, while 6% of women received this care from a doctor or clinical officer. Approximately 6% of women received the first postnatal check-up from a traditional birth attendant, while 3% and 1% of women received their first postnatal check-up from a patient attendant and other providers, respectively.

### Bivariate Results of Chi-Square Tests

Women consistently significantly differed (*P*  < .05) in level of utilisation of ANC, delivery care and postnatal care provided by a skilled health attendant when comparing them within different categories of education and wealth status (Table 2). Marital status was a significant factor with respect to women using both ANC and postnatal care provided by a skilled health attendant. Religion exclusively influenced use of ANC, while age, residence and ethnicity significantly affected utilisation of delivery care.

**Table 2:** Bivariate Results of Chi-Square Tests: Use of Maternal Healthcare Provided by a Skilled Health Attendant

**Variable**	**ANC**		**Delivery Care**		**Postnatal Care**		**Number of Women**
	**%**	***P*** **Value**	**%**	***P*** **Value**	**%**	***P*** **Value**	
Mother’s age at birth		.171		.007		.140	
15-19	84.4		78.8		42.4		33
20-34	95.2		78.4		58.3		453
35-49	88.9		64.8		52.8		125
Marital status		.041		0.445		.013	
Not married	88.2		77.4		62.9		221
Married	93.1		74.6		52.6		390
Residence		.343		.003		.344	
Rural	90.5		71.8		58.8		390
Urban	92.8		82.4		54.9		221
Education		<.001		<.001		<.001	
No education	80.4		62.9		42.3		97
Primary	92.0		74.5		55.2		388
Secondary and above	97.6		88.9		70.6		126
Work status		.222		.923		.950	
Not working	93.0		75.8		56.4		244
Working	90.2		75.5		56.1		367
Wealth quintile		<.001		<.001		.003	
Poorest	82.2		68.1		51.9		135
Poorer	92.9		71.4		51.8		112
Middle	90.4		61.7		44.7		94
Richer	95.5		77.2		59.8		92
Richest	96.6		90.4		66.9		178
Ethnicity		.525		.022		.073	
Chewa	90.6		73.0		53.6		481
Yao	90.3		87.1		67.7		31
Ngoni	93.3		80.0		62.2		45
Other	96.3		88.9		68.5		54
Religion		.027		.525		.710	
Catholic	91.6		79.0		61.3		119
C.C.A.P	97.2		78.0		53.2		141
Seventh-Day Advent/Baptist	95.5		68.2		56.8		44
Muslim	89.3		78.6		60.7		28
Other	88.1		73.6		55.2		277

Abbreviations: ANC, Antenatal care; C.C.A.P., Church of Central Africa Presbyterian.

### Predictors of Utilisation of Skilled Maternal Healthcare

Results for binary logistic regression analysis are presented in Table 3. As previously outlined, a maternal healthcare index was created to assess the continuum of maternal healthcare. Of all eight independent variables of the study, 3 variables, women’s residence (*P* = .006), education (*P* = .004), and wealth (*P* = .018) were significant predictors of utilisation of maternal healthcare provided by a skilled attendant. Urban women were less likely (odds ratio [OR] = 0.47,*P* = .006,95% CI *=* 0.28–0.81) to receive a continuum of maternal healthcare from a skilled health attendant compared to rural women. Similarly, women with less education (OR = 0.32,*P* = .001,95% CI = 0.16–0.64), and poor women (OR = 0.50,*P* = .04,95% CI = 0.26–0.97) were less likely to receive a continuum of maternal healthcare from a skilled health attendant.

**Table 3:** Binary Logistic Regression of Use of Skilled Attendance for Maternal Health Services

**Variable**	**ANC**		**Delivery Care**		**Postnatal Care**		**Maternal Healthcare Index**	
	**OR (95% CI)**	***P*** **Value**	**OR (95% CI)**	***P*** **Value**	**OR (95% CI)**	***P*** **Value**	**OR (95% CI)**	***P*** **Value**
Mother’s age at birth		.281		.143		0.383		.329
15-19 (ref)								
20-34	2.179 (0.718-6.614)	.169	0.812 (0.331-1.989)	.648	1.664 (0.792-3.495)	.179	0.603 (0.262-1.384)	.233
35-49	2.756 (0.782-9.709)	.115	0.516 (0.198-1.345)	.176	1.721 (0.763-3.882)	.191	1.073 (0.686-1.679)	.757
Marital status		.051		.705		.013		.106
Not married (ref)								
Married	1.849 (0.998-3.426)		0.923 (0.610-1.397)		0.639 (0.448-0.911)		1.335 (0.941-1.895)	
Residence		.105		.185		.012		.006
Rural (ref)								
Urban	2.104 (0.857-5.165)		1.500 (0.824-2.732)		1.944 (1.154-3.274)		0.475(0.280-0.808)	
Education		.016		.466		.016		.004
No education (ref)								
Primary	2.773 (1.301-5.908)	.008	1.318 (0.775-2.242)	.308	1.761 (1.067-2.908)	.027	0.320 (0.160-0.639)	.001
Secondary and above	5.071 (1.118-23.001)	.035	1.657 (0.702-3.912)	.249	2.727 (1.366-5.442)	.004	0.667 (0.401-1.109)	.118
Work status		.432		.890		.939		.871
Not working (ref)								
Working	0.772 (0.405-1.471)		1.029 (0.689-1.535)		0.987 (0.700-1.392)		0.927 (0.690-1.369)	
Wealth quintile		.022		<.001		.084		.018
Poorest (ref)								
Poorer	2.326 (0.956-5.661)	.063	1.109 (0.632-1.946)	.717	1.012 (0.602-1.699)	.965	0.503 (0.259-0.975)	.042
Middle	1.604 (0.670-3.839)	.288	0.673 (0.380-1.192)	.174	0.685 (0.395-1.189)	.178	0.598 (0.302-1.184)	.140
Richer	3.407 (1.076-10.787)	.037	1.653 (0.836-3.270)	.149	1.414 (0.777-2.572)	.256	0.327 (0.166-0.645)	.001
Richest	8.973 (2.216-36.342)	.002	4.736 (1.984-11.304)	.000	1.750 (0.901-3.396)	.098	0.786 (0.433-1.426)	.428
Ethnicity		.756		.842		.487		.515
Chewa (ref)								
Yao	0.394 (0.051-3.053)	.372	1.821 (0.385-8.610)	.449	1.735 (0.588-5.120)	.318	0.644 (0.320-1.297)	.218
Ngoni	0.594 (0.144-2.457)	.472	0.891 (0.370-1.146)	.796	1.353 (0.666-2.752)	.403	0.842 (0.275-2.581)	.764
Other	0.989 (0.186-0.5262)	.990	1.199 (0.31-3.332)	.728	1.602 (0.781-3.286)	.198	0.980 (0.420-2.289)	.964
Religion		.129		.650		.676		.948
Catholic (ref)								
C.C.A.P.	2.679 (0.784-9.160)	.116	0.875 (0.468-1.637)	.676	0.673 (0.400-1.132)	.135	1.078 (0.680-1.709)	.749
Seventh-Day Advent/Baptist	3.749 (0.694-20.258)	.126	0.600 (0.260-1.383)	.230	0.891 (0.423-1.875)	.761	0.873 (0.565-1.350)	.542
Muslim	1.100 (0.135-8.988)	.929	0.425 (0.103-1.745)	.235	0.796 (0.261-2.425)	.687	1.021 (0.523-1.992)	.953
Other	0.859 (0.376-1.965)	.719	0.847 (0.485-1.497)	.559	0.826 (0.518-1.317)	.422	1.089 (0.376-3.154)	.875

Abbreviations: ANC, Antenatal care; C.C.A.P., Church of Central Africa Presbyterian; OR, odds ratio. Note: The maternal healthcare index indicates the continuum of care, and is based on combining data for utilisation of antenatal care, delivery care and postnatal care provided by a skilled attendant.

## Discussion

The aim of this study was to identify which individual factors best predict utilisation of skilled maternal healthcare in a sample of women residing in Lilongwe district of Malawi. While the discussion of the findings presented below is by no means exhaustive, it aims to address several issues arising from the research in relation to a review of the literature.

In total, 91% of women reported obtaining ANC from skilled providers, indicating high use of skilled ANC services in Lilongwe, as previously reported in other studies.^45,51,52^ However, among women who visited an ANC clinic, only 50% visited at least four times or more during their last pregnancy. Furthermore, only 11% of women reported their first ANC visit as having occurred within the first trimester. Although use of ANC was therefore high, many women did not receive at least four ANC skilled assessments,and neither did they receive ANC assessments as early as possible during the first trimester, as recommended by the WHO^53^ and Malawi’s Ministry of Health.^54^ These findings indicate that women are not receiving comprehensive ANC services, signifying critical gaps in utilisation of ANC services.

While the study found a high utilisation rate of skilled ANC, a relatively low proportion (76%) of deliveries were conducted by a skilled attendant. This finding is consistent with several studies that indicate that receiving ANC from a skilled attendant does not in itself guarantee that women will seek and receive skilled delivery care.^13,15,18,55,56^ Low utilisation of skilled attendants especially during delivery in LMICs has been reported by several other researchers.^13,15,18,55^

A low utilisation rate of skilled attendance during postnatal care was found, with 34% of women reporting not receiving any postnatal check-up. This study also highlights a very low utilisation rate (32%) of skilled attendance for postnatal care during the first hour after birth. This finding is consistent with those of several other studies conducted in LMICs, which indicate very low coverage levels of postnatal care.^13,14,18,55,57^ Because more women in LMICs do not give birth in a health facility, this poses challenges to access to postnatal care for women and newborns.^58^

Of all eight independent variables of this study, the variables of residence, education, and wealth were significant predictors of utilisation of maternal healthcare provided by a skilled health attendant. Similarly, Yaya et al^59^ reported that wealth, education and residence (urban vs. rural) had a significant effect on uptake of maternal healthcare in Malawi; although notably their study found that women in rural areas were less likely to receive four antenatal visits, skilled birth attendance, and postnatal care. Results of the present study indicate that urban women were less likely than rural women to receive a continuum of maternal healthcare from a skilled health attendant. This finding is contrary to several studies that report that generally urban women are more likely to use maternal health services than rural women.^19,60,61^ There is a need for further research to investigate this finding. However, as specified by Lungu et al,^62^ some cohorts in urban areas, including children living in urban slums, do not experience an urban health advantage, but rather “in the context of increasing urbanisation and urban poverty manifesting with proliferation of urban slums, the health of under-five children in slum areas remains a public health imperative in Malawi” (p. 1). At any rate, decreasing newborn and maternal deaths necessitates equitable distribution of resources in both urban and hard to reach districts.^63^

Findings also indicated that less educated and poor women were less likely to receive a continuum of maternal health services from a skilled health attendant. Comparable to these findings, in a study assessing the continuum of maternal healthcare in South Asia and sub-Saharan Africa using DHS data from nine countries including Malawi, Singh et al^64^ found that only a small subsection of women reported receiving all elements of the continuum of care, and these women tended to be the most educated and richest, alongside women with a high amount of autonomy. Mothers who are more educated are more likely to seek prenatal care, birth attendance by trained medical staff, immunisation and modern healthcare for their young children.^65^ Similar to this study’s findings, in a multi-country study of utilisation of maternal health services in Africa, Tsala Dimbuene et al^66^ found that women residing in higher socioeconomic households had greater access to and utilisation of maternal healthcare.

Findings of this study therefore indicated that residence, education, and wealth were significant predictors of utilisation of ANC, delivery care, and postnatal care provided by a skilled attendant. With regards to ANC, Chimatiro and colleagues^67^ comparably reported that, in Malawi, lack of knowledge on the importance of ANC during the first 3 months of pregnancy was a key barrier to attending clinics, and recommended information, education, and communication on ANC for women. Studies conducted elsewhere report that women fail to visit ANC clinics due to lack of information on the content, schedule and advantages of ANC.^25,68^ Failure to visit ANC services by pregnant women in Malawi may also be influenced by pregnancy-associated beliefs, notably witchcraft.^69^ Other factors may include lack of money.^70^ As a crucial link in the continuum of care, ANC offers significant opportunities to reach women with effective clinical and health promotion interventions.^71^

With regards to delivery care, Katenga-Kaunda^23^ similarly reported that, in Northern Malawi, women with a higher socioeconomic status and those with higher education were more likely to deliver at a health facility. Machira and Palamuleni^72^ reported lack of money for transport to access healthcare facilities as a factor resulting in non-institutional childbirth in Malawi. A critical strategy to reduce maternal morbidity and mortality is the assistance at delivery by skilled health personnel at every birth.^73,74^ Evidence suggests that women who attend four or more ANC visits and deliver in a health facility in the presence of a skilled birth attendant are more likely to receive immediate skilled postnatal care.^55,57^

With respect to postnatal care, comparable to this study’s findings, Zamawe et al^75^ reported that, in Malawi, lack of knowledge of postnatal care was a critical barrier to care, and recommended extensive maternal health education programmes. Postnatal care is essential for both the mother and infant as it enables health practitioners to provide prompt treatment for complications arising from the delivery as well as providing the mother with important information on caring for herself and her baby.^45^ Postnatal care, particularly within the first 24 hours after childbirth, is critical to the health and survival of a mother and her newborn.^2,58^

## Limitations

A limitation of this study is that a sub-sample of 2010 MDHS data was used, ie, women residing in Lilongwe. Furthermore, the study used secondary data from the 2010 MDHS, although a more recent MDHS is now published.^1^ However, the 2010 MDHS was the most recent available at the time that the study was conducted. It is also noteworthy that while the study comprised eight independent variables derived from the MDHS, additional individual behavioural factors such as problem-solving, being task-focused, or seeking social support may be influential at the individual level for health service utilisation.

A cross-sectional design was used for this study. While a longitudinal design allows participants to be assessed and trends monitored across several time points, a limitation of a cross-sectional design is the inability to assess trends over time; indeed, only an association and not causation may be inferred from cross-sectional studies.^76^ However, cross-sectional studies are appropriate for assessing the prevalence of behaviours or diseases in a given population,^76^ and therefore the use of cross-sectional data for the present study was suitable in terms of identifying women who had received maternal healthcare from a skilled attendant.

It is also possible that MDHS respondents may have experienced recall bias, which may be defined as “systematic error due to differences in accuracy or completeness of recall to memory of past events or experiences” (p. 240).^77^ In the present study, women reported in the MDHS if they received maternal healthcare from a skilled attendant for their most recent live birth within the 5 years preceding the survey. This interval of 5 years may therefore have impacted on respondents’ accuracy of recall when completing the questionnaire.

With respect to the conceptual framework used in this study, whilst acknowledging the strengths and contributions of Andersen’s model in health services research, it is worth noting that the model has some potential weaknesses and criticisms. Several researchers have expressed concern about the validity of the study concepts, specification and testing of the hypothesised relationships, and the robustness and generalisability of the findings based on the model.^78,79^ Other critics have noted that the model has failed to include genetics and psychosocial components (eg, health beliefs and knowledge regarding illness) and has ignored the broader social contexts (such as social networks) in which individuals decide to seek healthcare.^80^ Andersen,^81^ however, argued that social structure is included in the predisposing characteristics component. Another general criticism is that the wide range of variables and differing levels of analysis included renders it difficult to collect data to test the complete model.^39^

## Conclusion

Continuity or the “continuum of care” is the central principle underpinning maternal, newborn and child health programmes.^2^ As suggested by Singh et al^64^ (p. 6), “each element of the continuum of care for maternal health provides essential and potentially lifesaving services.” Access to skilled health providers for antenatal, delivery and postnatal care enables early detection of complications for mothers and newborns, and allows prompt treatment, as well as timely referral to a facility where a complication can be appropriately managed, besides being cost-effective and feasible in resource-poor countries.^45,82^ All women should have access to a skilled health practitioner during pregnancy, childbirth and the postnatal period to reduce maternal and neonatal deaths.^2^

Findings of this study indicated high utilisation of skilled ANC services among women in Lilongwe. However, moving along the continuum of care, use of skilled care was strikingly lower for delivery care and postnatal care, indicating that use of skilled ANC does not in itself guarantee that women will use skilled delivery care and postnatal care. This calls therefore for the development of policy and interventions that facilitate a “continuum of care” for maternal health services from ANC, delivery care to postnatal care.

Furthermore, the findings indicate that women with high education and income status are more likely to receive a continuum of maternal healthcare from a skilled health attendant. Policies and programmes should therefore aim to increase utilisation of skilled maternal healthcare for women with less education and poor women. While this study aimed to identify predictors of utilisation of skilled maternal healthcare, it is important that future qualitative research is conducted to provide a better understanding of such inequalities regarding skilled maternal healthcare amongst women residing in Lilongwe.

Strengthening education for girls (SDG goal 4) in southern Africa would improve maternal health outcomes (part of goal 3).^32^ This reflects the association between high-quality education and better health.^30^ Supporting education for girls and women, including for those with less wealth, would therefore strengthen maternal healthcare utilisation in Malawi. In relation to the SDGs, Nilsson et al^32^ (p. 321) suggest that, “if mutually reinforcing actions are taken and trade-offs minimised, the agenda will be able to deliver on its potential … The importance of such interactions is built into the SDGs: ‘policy coherence’ is one of the targets.” Malawian policies therefore require policy coherence, to strengthen education and economic empowerment for girls and women, and by extension, utilisation of skilled maternal healthcare.

## Acknowledgements

We wish to extend our gratitude to Irish Aid for providing the primary researcher (IK) with a bursary for PhD research and training for the International Doctorate in Global Health at the Centre for Global Health, Trinity College Dublin, Dublin 2, Ireland. We would also like to thank the Health Research Capacity Strengthening Initiative (HRCSI) by the National Commission for Science and Technology in Malawi for providing the primary researcher with a bursary for PhD data collection.

## Ethical issues

Ethical approval for this study was granted by the Health Policy and Management/Centre for Global Health Research Ethics Committee at Trinity College Dublin, Ireland, and the College of Medicine Research Ethics Committee (COMREC) at University of Malawi, Zomba, Malawi.

## Competing interests

Authors declare that they have no competing interests.

## Authors’ contributions

IK conducted the statistical analysis as part of her doctoral research, with the supervision of ACM, HM, and MM. IK wrote the first draft of the manuscript; JM edited the manuscript. All authors contributed to manuscript revision, read, and approved the submitted version.

## Authors’ affiliations

^1^School of Public Health and Family Medicine, College of Medicine, University of Malawi, Blantyre, Malawi. ^2^Centre for Social Research, Chancellor College, University of Malawi, Zomba, Malawi. ^3^Department of Psychology, Maynooth University, Maynooth, Ireland. ^4^Assisting Living and Learning (ALL) Institute, Maynooth University, Maynooth, Ireland. ^5^School of Nursing, Midwifery and Health Systems, University College Dublin, Dublin, Ireland. ^6^Centre for Rehabilitation Studies, Stellenbosch University, Cape Town, South Africa. ^7^Olomouc University Social Health Institute, Palacký University, Olomouc, Czech Republic.

## Endnotes

[1] A health surveillance assistant is the lowest cadre in the Ministry of Health based at the community level and his or her catchment area comprises 1000 people. This cadre is mainly involved in health promotion and preventive health services including conducting health talks at facilities as well as during outreach.

## Key messages

**Implications for policy makers**

- Use of skilled antenatal care (ANC) does not in itself guarantee that women will use skilled delivery care and postnatal care. This calls therefore for the development of policies and interventions that facilitate a “continuum of care” for maternal health services from ANC, delivery care to postnatal care.
- Policies and programmes should aim to increase utilisation of skilled maternal healthcare for women with less education and low-income status.
- Policies should focus on strengthening education and economic empowerment for girls and women to improve maternal healthcare utilisation.

**Implications for public**

While Malawi has made significant improvements regarding child mortality, maternal and newborn mortality rates remain too high, with Malawi having one of the highest maternal mortality ratios globally. The aim of this study was to identify which individual factors best predict utilisation of skilled maternal healthcare in a sample of women residing in Lilongwe district of Malawi. Identifying which of these factors play a significant role in determining utilisation of skilled maternal healthcare is required to inform policies and programmes in the interest of achieving increased utilisation of skilled maternal healthcare in Malawi. Urban women, women with less education, and poor women were less likely to use a continuum of maternal healthcare from a skilled health attendant. Policies and programmes should aim to increase utilisation of skilled maternal healthcare for women with less education and low-income status by promoting education and economic empowerment initiatives for girls and women.
